# Theoretical Basis and Application for Measuring Pork Loin Drip Loss Using Microwave Spectroscopy

**DOI:** 10.3390/s16020182

**Published:** 2016-02-02

**Authors:** Alex Mason, Badr Abdullah, Magomed Muradov, Olga Korostynska, Ahmed Al-Shamma’a, Stefania Gudrun Bjarnadottir, Kathrine Lunde, Ole Alvseike

**Affiliations:** 1 Faculty of Engineering and Technology, Liverpool John Moores University, Henry Cotton Building, 15-21 Webster Street, Liverpool L3 2ET, UK; B.M.Abdullah@ljmu.ac.uk (B.A.); M.Muradov@2009.ljmu.ac.uk (M.M.); O.Korostynska@ljmu.ac.uk (O.K.); A.I.Al-Shamma'a@ljmu.ac.uk (A.A.-S.); 2 ANIMALIA, Norwegian Meat and Poultry Research Centre, Lørenveien 38, Postboks 396 Økern, Oslo 0513, Norway; stefania.bjarnadottir@animalia.no (S.G.B.); kathrine.lunde@animalia.no (K.L.); ole.alvseike@animalia.no (O.A.)

**Keywords:** drip loss, microwave, sensor, water holding capacity, pork loin, meat processing

## Abstract

During cutting and processing of meat, the loss of water is critical in determining both product quality and value. From the point of slaughter until packaging, water is lost due to the hanging, movement, handling, and cutting of the carcass, with every 1% of lost water having the potential to cost a large meat processing plant somewhere in the region of €50,000 per day. Currently the options for monitoring the loss of water from meat, or determining its drip loss, are limited to destructive tests which take 24–72 h to complete. This paper presents results from work which has led to the development of a novel microwave cavity sensor capable of providing an indication of drip loss within 6 min, while demonstrating good correlation with the well-known EZ-Driploss method (R^2^ = 0.896).

## 1. Introduction

From the point at which an animal is slaughtered during the meat production process, it is inevitable that water will be lost from the carcass. This is a key concern for meat producers as this water content is said to contribute to the juiciness and tenderness of meat products [1,2], which impacts on consumer opinion, thus affecting demand and saleable value. While the loss of product quality and appeal is often difficult to measure due to its subjective nature, Table 1 demonstrates the average basic constituents of common meat products, with many containing greater than 75% water [3].

Since most meat products are sold on the basis of their weight, it stands to reason that loss of water is directly proportional to a loss in revenue. It is estimated that for large production facilities (*i.e.*, those processing in the order of thousands of animals per day), for every 1% of water lost, this could equate to €50,000 (this estimate is based up on a large processing plant, but of course is dependent on the volume of meat trimmings produced in addition to the market value of meat at the time of processing) per day in lost revenue. For this reason, meat producers are keen to employ new tools which enable them to monitor the production process more quickly and effectively than current methods allow, therefore permitting minimization of water loss. This paper builds significantly upon previous proof of concept work [4,5] by the authors in the use of a novel microwave spectroscopy technique to measure drip loss in pork. In particular, this paper demonstrates how the technique compares with the most widely used industry method for drip loss measurement (*i.e.*, the EZ-Driploss method).

**Table 1:** Average constituents of common meat cuts.

Meat	Cut	Water %	Protein %	Fat %	Ash %
Pork	Boston butt	74.9	19.5	4.7	1.1
	Loin	75.3	21.1	2.4	1.2
	Cutlets/chops ^1^	54.5	15.5	29.4	0.8
	Ham	75.0	20.2	3.6	1.1
	Side cuts	60.3	17.8	21.1	0.85
Beef	Shank	76.4	21.8	0.7	1.2
	Sirloin steak ^1^	76.4	21.8	0.7	1.2
Chicken	Hind leg	73.3	20.0	5.5	1.2
	Breast	74.4	23.3	1.2	1.1

^1^ With adhering adipose tissue.

## 2. Water Holding Capacity

The term drip loss, or its reciprocal parameter Water Holding Capacity (WHC), is used by the food industry to refer to the ability of meat products to retain water, with much of our current knowledge on the topic being based on fundamental research by Hamm [6] in 1960, followed by Offer and Trinick [7] in 1983. There is a general agreement [8,9] that water in meat can exist in three forms: (1) bound; (2) immobilized; and (3) free, with these representing as much as 5%, 15%, and 85% of total water content, respectively. Bound water is tightly bound to proteins and is not free to move around, cannot be frozen, and is not affected by chemical changes (e.g., pH), while immobilized water shares similar properties, albeit with weaker protein bonding. Free water, on the other hand, is held loosely in the capillary space between and within proteins and, unlike bound and immobilized water, is easily lost. Therefore, anything which alters the protein structure or spacing will affect the ability of the meat to retain free water. Examples of factors which might impact protein structure, and hence drip loss, include *post mortem* rigor (the steric effect) [10,11]; pre-slaughter stress [8,12]; pH [1,13]; and common processing techniques [14], such as heating, grinding, cutting, pressing, and freezing.

Measuring the drip loss of meat at various stages during the production process could enable impact assessments of the factors causing water loss. In principle this would allow optimization of processes in addition to the sorting of carcasses or cuts so that resources are allocated to optimal products. Such action would reduce lost revenue and ensure consistent meat quality and tenderness. In practice, however, measurement of drip loss is challenging, as the current commercially available methods are destructive, manual, and time consuming. Furthermore, despite efforts described by Honikel [15], there is no international standard method, which makes comparison of results derived from the various techniques difficult; Table 2 presents an overview of these techniques.

A number of attempts to provide a sensor technique to standardize and automate the measurement of drip loss or WHC have been made. X-ray diffraction has been used extensively within the food industry for foreign object detection (e.g., metal, glass, and plastic shards) in addition to recent systems such as the MeatMaster (FOSS, Denmark) which can give online prediction of fat content in meat trimmings. It has also proven to give excellent resolution in relation to the spacing between muscle filaments [16,17,18]; since it is said that most of the water in meat is held between the muscle filaments (or myofibrils) this can give an indication of WHC of samples. Despite this, X-ray systems have been unable to demonstrate a method for online prediction of WHC, most likely because the technique, itself, is exacting, requiring careful dissection of muscle slips and long exposure times. Notably, however, recent work from O’Farrell *et al.* [19] has demonstrated an energy-dispersive diffraction system with a correlation of R^2^ = 0.72 when compared with the industry standard EZ-Driploss method which is described in Table 2. While this is promising, particularly because the technique offers measurement speeds of minutes rather than hours as described in some works, X-ray systems are usually deployed at only one or two locations across a production line (e.g., at the point of packaging if checking for foreign objects). For effective drip loss measurement, many points of measurement are required which precludes the use of X-ray largely due to the costly nature of the equipment in addition to concerns regarding worker exposure to radiation.

Near-Infrared Spectroscopy (NIRS) has also been considered in relation to the issue of drip loss. As with X-ray systems, NIRS devices are becoming popular in the meat industry for online compositional analysis; three examples are the QVision 500 Analyzer (TOMRA, Norway), ProFoss (Foss, Norway), and Spektron (Prediktor, Norway). However, work by Kapper [20] and O’Farrell *et al.* [19] have demonstrated poor correlation between NIRS measurements and drip loss; Kapper noted R^2^ between 0.36 and 0.73 depending on meat color and O’Farrell’s work demonstrated R^2^ = 0.47, despite a number of outlier data points (10%) being removed in the latter work.

A further method has also been demonstrated by Lee *et al.* [21] using an electrical conductivity measurement to attempt correlation of drip loss. This method showed some promise, with three categories of drip loss used to determine the effectiveness of the technique: <2%, 2%–6%, and >6%. It was demonstrated that 80% of the time the technique could correctly categorize meat samples from a production plant, albeit under laboratory conditions. It is notable however that in the vast majority of production facilities, the invasive nature of the electrodes used in this method would preclude it from online testing due to the potential for product contamination and spoilage.

**Table 2:** Manual methods of measuring water holding capacity, or its reciprocal parameter, drip loss, used in the commercial and research environments.

Method	Description
**EZ-Driploss**	The day after slaughter the muscles to be analyzed are taken from the carcass. Within one hour, a 25 mm slice is cut at a right angle to the muscle fiber direction, with samples being taken from this slice using a cork borer, again cutting in the fiber direction. The cylindrical sample, 25 mm in diameter and 25 mm in height, is weighed and then placed in a special container equipped with a lid to avoid evaporation and loss of water. The container is stored for 24 h at 4 °C–6 °C before being weighed again; the WHC is determined by ratio of the two weight measurements [22].
**Filter Paper Press**	This method involves the pressing of a meat sample into a filter paper; typically a defined pressure is recommended and the amount of released water is determined by weighing the meat sample or the filter paper before and after pressing. Hamm suggested a more rigorous protocol in 1972, which involves small meat samples (0.3 g) being pressed onto a filter paper at a pressure of 35 kg/cm^2^ between two plates. Five minutes later, meat samples are removed. The areas covered by the flattened meat sample and the stain from the meat sample are marked and measured [23,24].
**Centrifuge**	A weighed meat sample (3–4 g) is centrifuged at 100,000 *xg* for 1 h in a stainless steel tube. The water released from the meat is decanted off as quickly as possible (in order to avoid re-absorption). The meat sample is removed from the tube with forceps, dried with tissue paper, and then reweighed to determine liquid loss. If the residue is dried in the tube at 105 °C, the total water content of the sample can be determined, and WHC can be expressed as released or bound water as a percentage of total water. The need for a high-speed centrifuge makes it almost impossible to use this type of method in a slaughterhouse [25].
**Bag**	Meat samples (weighing approxiamte 100 g) are cut from a carcass and immediately weighed. The samples are then placed in a bag and hung in an airtight container using a hook under the lid. After the required storage time at the temperature under investigation (usually 24–48 h at 1 °C–4 °C) samples are weighed again [15].
**Absorption**	Cotton-rayon material is inserted into a “+” shaped incision in the longissimus muscle through the subcutaneous fat layer. The incision is approx. 2.4 inches deep at a well-defined place (e.g., 12th rib) and is left for either 15 min at 15 min post-mortem or 15 min at 24 h post-mortem. Absorption is calculated as the difference between the final weight plus exudates and the initial dry weight of the material. Notably this technique is the quickest of all those listed here, however it also requires a skilled operator to enable repeatable incisions and measurement [26].

## 3. Microwave Spectroscopy

Sensors which operate at microwave frequencies are widely used in a variety of industrial sectors in addition to having been demonstrated in the research domain. Examples include structural analysis [27,28], water quality monitoring [29,30,31], and medical applications [32,33,34,35,36,37]. Aside from research considering quality classification of fresh [38,39] and cured meats [40,41], there is little evidence of microwave sensors making a significant impact in the food industry. This point is supported by a recent comprehensive review of electromagnetic wave sensors (from radio frequencies to X-ray) conducted by Damez and Clerjon [42].

Microwave sensors provide the opportunity for a rapid non-invasive and robust method of materials analysis. The authors have also demonstrated that the technique can take on many physical forms, including resonant cavities [43], planar structures [30,37], and fluidic devices [36], which makes it highly adaptable to a range of situations and applications. Furthermore, the technology to generate and detect microwave signals is inexpensive; it is featured in the many millions of smart phone devices, tablets, and portable computers, for example, that make use of wireless communications. This is a particularly attractive feature for the food industry, given the desire for high-resolution drip loss information from across production facilities, which is unlikely to be cost effective with technologies such as X-ray. Added to this, the technique is non-ionizing, utilizing less than 10 mW of power, significantly less than modern wireless communication devices and is, therefore, thought to be safe to use within food production without fear of harming the product or nearby workers.

The principle of monitoring using microwave sensors, in the context of this work, is based on the interaction of electromagnetic (EM) waves with a sample under test. When this sample is exposed to EM irradiation it alters the velocity of the signal, attenuates, or reflects it. If one considers a hollow structure with conducting walls (*i.e.*, a cavity), it will resonate when it is excited at an appropriate EM frequency provided some means for this to occur is introduced, for example, via a small antenna placed inside it. Resonant modes occur inside the cavity when the electric or magnetic components of the EM signal form standing waves, which are dependent on the dimensions of the cavity and the dielectric properties of the test sample. The resonant frequency for TE_nml_ and TM_nml_ modes in a rectangular waveguide [44] can be calculated using Equation (1), where *c* is the speed of light, *µ_r_* is of the relative permeability, *ε_r_* is the relative permittivity, *p_nm_* is the value of the Bessel function for the TE or TM modes of a rectangular waveguide, *a* is the width of the cavity, *b* is the height of the cavity and *d* is the depth of the cavity.

(1) $$f_{mnl}=\frac{c}{2\pi \sqrt{\mu_{r}\varepsilon_{r}}}\sqrt{{\left(\frac{m\lambda }{a}\right)}^{2}+{\left(\frac{n\lambda }{b}\right)}^{2}+{\left(\frac{l\pi }{d}\right)}^{2}}$$

Any number of such antennae may be placed within the cavity for the purposes of transmission and reception of EM energy, however the most typical configurations involve one and two port (thus, one or two antennae) cavities since often the materials placed within them are assumed to be relatively homogeneous and therefore further ports serve little purpose.

**Figure 1:**
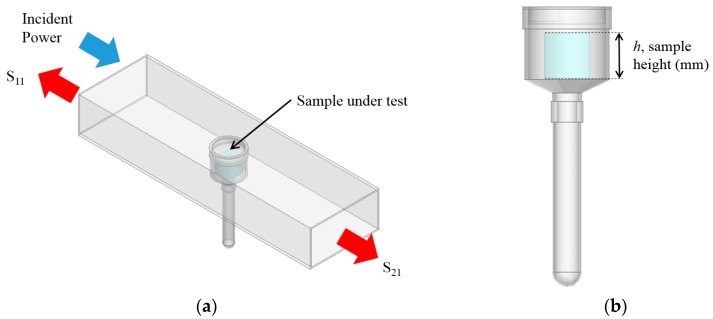
(**a**) A 3D model of the cavity designed for this work; and (**b**) the modelled EZ-Driploss sample container which is used to hold meat samples during the measurement procedure.

In a one port configuration it is possible, using a Vector Network Analyzer (VNA), to measure the power which is reflected from the cavity; this is often referred to simply as an S_11_ measurement. In a two port configuration, one can also measure power transmitted through the cavity; this is referred to as an S_21_ measurement. This is illustrated in Figure 1a, which shows a 3D model of the cavity designed specifically for this work using Ansys High Frequency Structure Simulator (HFSS) finite element modelling package; Figure 1b shows the sample model which is an EZ-Driploss sample container. With reference to the full description of this drip loss measurement method given in Table 2, the EZ-Driploss container holds a cylindrical meat sample in the larger top section, with water lost over the 24 h measurement period being collected at the bottom of the thin tube. The cavity is designed such that only the larger top section resides within the cavity as it serves no purpose to measure any fluids lost from the meat samples. EZ-Driploss containers were used to hold the sample during the course of this work to allow direct correlation of the standard EZ-Driploss measurement against the data acquired from the microwave cavity.

**Figure 2:**
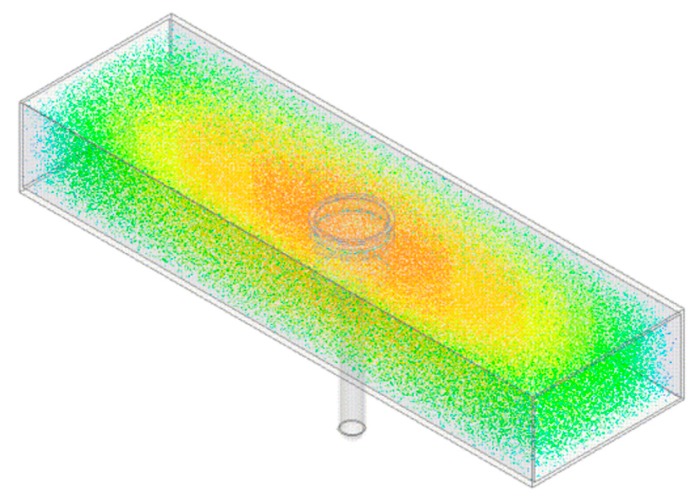
The resonant cavity model with electric field distribution overlay when resonant at approx. 1.5 GHz, which is where the TE_010_ mode is present within the cavity according to Equation (1). Notably the electric field is concentrated, as noted by the red/orange coloration, around the EZ-Driploss container which ensures maximum interaction of the EM signal with the target sample.

**Figure 3:**
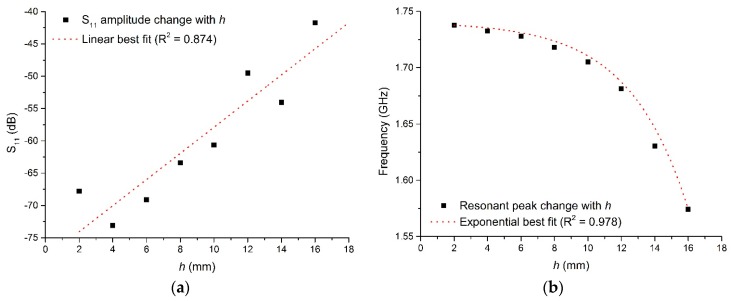
Cavity response when varying sample height, *h*, for (**a**) the S_11_ signal amplitude, and (**b**) the frequency at which the resonant TE_010_ mode occurs.

From Equation (1) it is shown that all EM modes have the same dependence upon $\sqrt{\varepsilon_{r}}$, so when the cavity is excited over an appropriate range of frequencies and the resultant spectra is captured, the resonant peaks corresponding to these modes will shift, typically in frequency and amplitude, as the permittivity is varied. This can be demonstrated by taking the model illustrated in Figure 1a and varying the sample height, *h*, shown in Figure 1b such that the sample in this case is water when the cavity it resonating in the TE_010_ mode, as represented in Figure 2. It is assumed that this approximates the composition of most fresh meat products immediately *post mortem* since, as noted in Table 1, water is the major constituent. Figure 3 shows the relationship for both the signal amplitude and resonant frequency shift as *h* is varied in the range 2–16 mm. A high correlation, using this modelling approach, is demonstrated for both signal amplitude (R^2^ = 0.874) and resonant frequency (R^2^ = 0.978) and shows the responsiveness of the technique to variations in water, which provides the basis for its use in monitoring the drip loss of meat samples.

## 4. Experimental Methodology

The purpose of the experimental work outlined in this paper was to compare and correlate drip loss measurements from the microwave cavity illustrated in Section 3 with the current dominant method used in industry, *i.e.,* the EZ-Driploss method [22].

To this end, a total of 24 pork carcasses were selected after pre-sorting based on a wide range of pH and breeds in an attempt to obtain a large variation of drip loss across the samples. While it was impossible to guarantee with absolute certainty a wide range of drip loss from the selected samples (*i.e.*, there is no current online method available for this purpose), the link between pH and ability of meat to retain water is widely reported [8]. Furthermore, the carcasses were selected from Noroc and Landrace breeds since there is typically a pH difference between them; namely, Landrace often have a lower pH than Noroc breeds.

To prepare the samples, a loin was taken from each carcass, with each loin having a slice of approximate 20 mm thickness taken from the middle. Each loin was split into two portions; one portion was retained by researchers at Animalia to establish a baseline or control EZ-Driploss measurement, and the other was provided to the researchers from Liverpool John Moores University who had developed the microwave cavity sensor. All work measurements took place simultaneously at Animalia’s pilot plant facility, located in Oslo, Norway.

**Figure 4:**
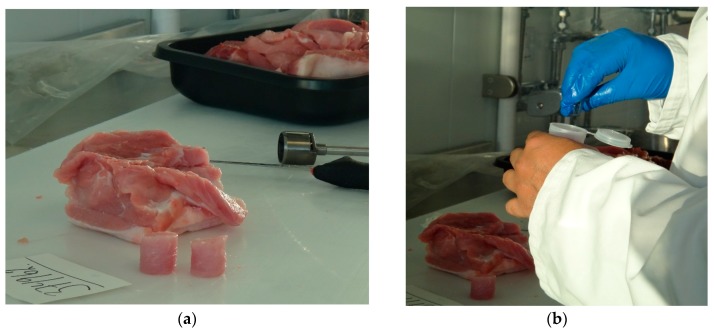
(**a**) Preparation of the 25 mm diameter core samples from the 20 mm thick slice of loin; and (**b**) placement of a sample in an EZ-Driploss container.

From these loin portions, two 25 mm diameter core samples were taken with a borer. Each core sample was then placed into a separate EZ-Driploss polypropylene container and lid was closed prior to measurement commencing. Therefore, four core samples were taken from each loin sample and measured using the EZ-Driploss method, giving 96 core samples in total. The procedure for sample preparation is illustrated in Figure 4. Care was taken when preparing samples to avoid deposits of fat and other visible inconsistences in the product, which is standard practice when employing the EZ-Driploss method.

All core samples were weighed prior to being stored for a period of 24 h at between 4 °C and 6 °C. After this period, all core samples were weighed once more and drip loss was calculated using Equation (2), where *W_c_* is the weight of an empty EZ-Driploss container, *W_t_* is the weight of the container with meat and exudate and *W_l_* is the weight of the container with liquid only. While only one of the core samples taken from each loin could be measured using the microwave method (therefore, 24 measurements in total), an average value was determined for the EZ-Driploss method from across the four core samples taken from each loin. All measurements (*i.e.*, 96 EZ-Driploss and 24 using the microwave cavity) took place over three days, which allowed time for system configuration between measurements and sample preparations.

(2) $$Drip\;Loss_{EZ}\left(\%\right)=\frac{W_{l}-W_{c}}{W_{t}-W_{c}}\times 100$$

**Figure 5:**
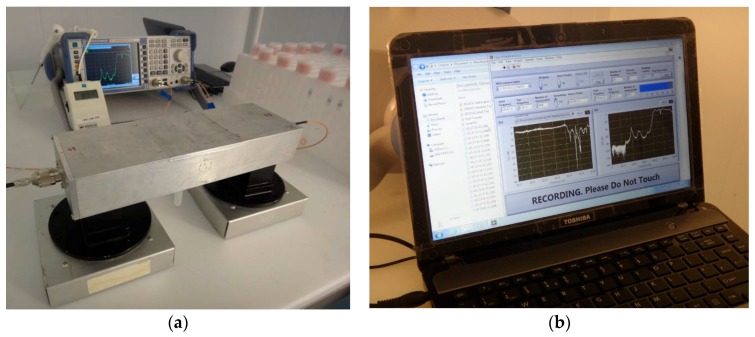
(**a**) Experimental setup showing the microwave cavity connected to a Rohde and Schwarz Vector Network Analyzer; and (**b**) a bespoke LabVIEW^®^ interface capturing spectral data.

The microwave cavity sensor was used immediately after sample preparation, with the sample being placed inside the cavity as shown in Figure 5a. The measurement equipment was configured inside a refrigerated chamber in order to ensure that measurements were undertaken at similar temperatures to which samples were stored. Both reflected (S_11_) and transmitted (S_21_) power measurements were taken for 30 min per sample; the microwave spectrum was captured between 1 and 6 GHz every minute. This timing was established as a result of a preliminary study [4] which measured similar samples over a 24 h period. A Rohde and Schwarz ZVL6 VNA was used for capturing spectral data, which was automated via a bespoke National Instruments LabVIEW^®^ interface as shown in Figure 5b.

## 5. Results and Discussion

### 5.1. Comparison of Drip Loss Measurements

**Figure 6:**
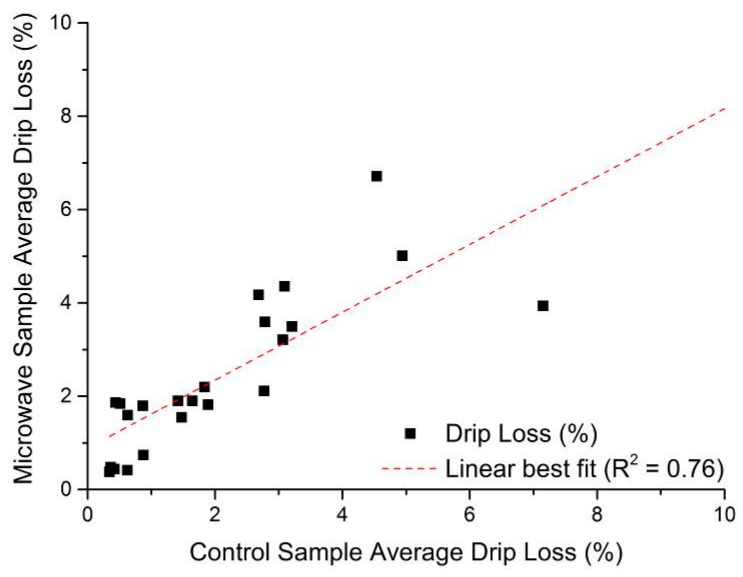
Correlation of drip loss in control sample and the portion of pork loin used for microwave measurement in this work, where R^2^ = 0.76.

As noted in Section 4, drip loss measurements following the EZ-Driploss method were conducted on two portions of the same meat, with one portion being used only for drip loss measurement and the other being used for measurements with the microwave cavity sensor. The comparison of EZ-Driploss measurements obtained is shown in Figure 6, and demonstrates that there is a reasonable agreement (R^2^ = 0.76) between the two portions of the same pork loin which gives confidence that the sample preparation methods are similar. It does however also serve to highlight the potential for error or variation when using the EZ-Driploss method since the meat itself is heterogeneous, and despite the best efforts of the operator, this is likely to be a factor. It is also notable that results from EZ-Driploss testing can vary from operator to operator, which is noted by a number of authors, including Christensen [45] for example. Therefore, when considering measurement data from the microwave sensor system, or any other for that matter, it is important to remember that the EZ-Driploss test, despite being a widely used and accepted method for drip loss measurement, harbors considerable inherent variability.

### 5.2. Microwave Cavity Measurements

**Figure 7:**
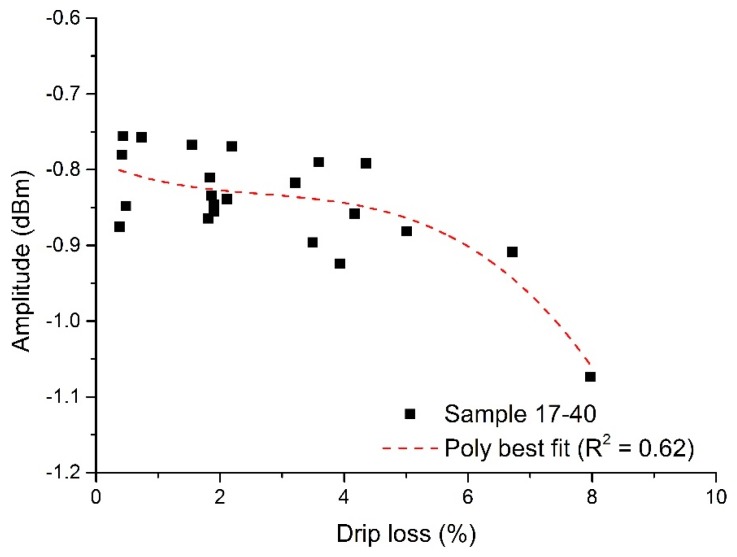
Correlation of the microwave cavity sensor at 4.23 GHz, with S_11_ amplitude changing as a function of drip loss (R^2^ = 0.62).

When using microwave sensors for measuring properties or changes in materials, it is often possible to derive a correlation directly from single measurements of the sample(s) under test and, therefore, one would be able to rapidly develop a calibration curve to define the sensor performance. However, due to the heterogeneous nature of meat and the small physical size of the sample, this typical approach yielded only a weak correlation at 4.23 GHz (S_11_), where R^2^ = 0.62. This is illustrated in Figure 7.

**Figure 8:**
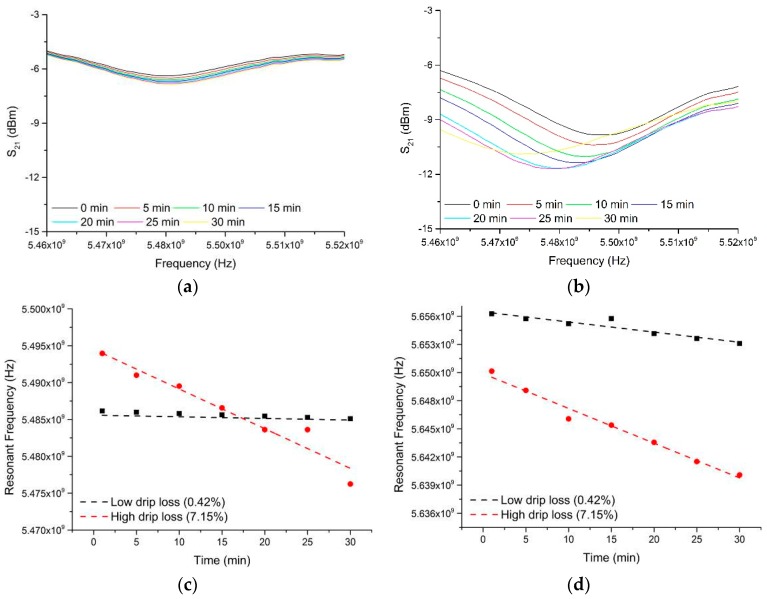
The microwave spectra between 5.46 and 5.52 GHz for two samples; one with (**a**) low drip loss (0.42%) and one with (**b**) high drip loss (7.15%) as determined by EZ-Driploss measurements. The change in resonant frequency in the range (**c**) 5.47 to 5.50 GHz and (**d**) 5.636 to 6.656 GHz are demonstrated and compared for the high and low drip loss samples.

Fortunately, data was collected for each sample over a time period of 30 min (where timing begins at the moment when the sample is inserted into the cavity sensor), with measurements taken at 1 min intervals. This gave the opportunity to consider whether the sensor responded to any immediate change in the sample after it was placed within the EZ-Driploss container. This yielded some rather interesting findings, as illustrated in Figure 8, whereby change in the S_21_ spectra gave a relationship to the end drip loss measurement for each sample. In particular, Figure 8 shows the microwave spectra between 5.4 and 6.0 GHz for two samples; one with a low drip loss (0.42%) and one with a high drip loss (7.15%) as determined by subsequent EZ-Driploss measurements. Over the 30 min measurement period both samples exhibited a reduction in resonant frequency, most notable in the range 5.47 to 5.50 GHz, as well as at 5.636 to 5.656 GHz. This reduction in resonant frequency is indicative of a change in the bulk relative permittivity of the meat sample, possibly due to diffusion or redistribution of water, post-preparation, while the sample is housed in the EZ-Driploss container. It is thought that such diffusion or redistribution of water would occur more rapidly in samples with a high drip loss owing to the availability of free water, thus enabling the sensor to be utilized for the purposes of determining the drip loss of the sample.

This principle was applied to all 24 samples measured using the microwave sensor technique, using the frequency shift gradient between the first and subsequent S_21_ measurements between 5.47 and 5.50 GHz as a reference against which to correlate with the EZ-Driploss results.

**Figure 9:**
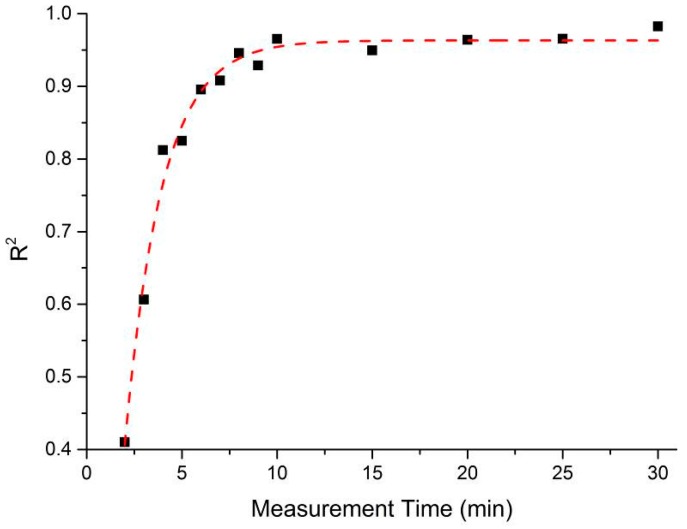
The relationship between R^2^ value and measurement time of the sample inside the microwave cavity sensor.

**Figure 10:**
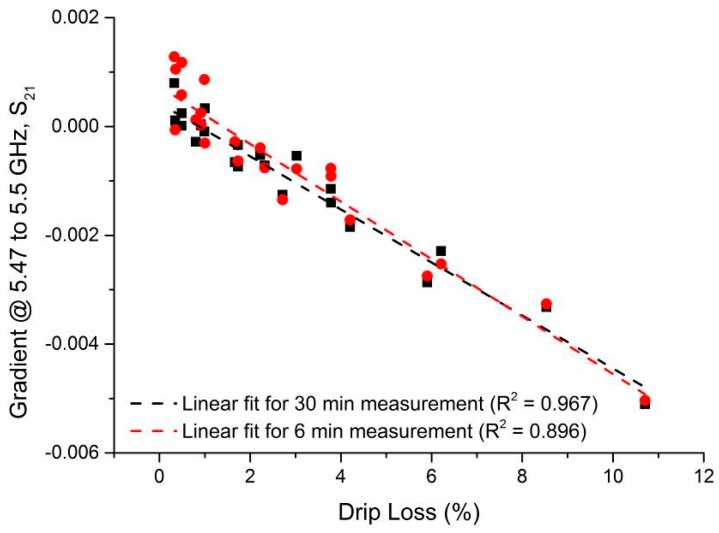
Correlation of the microwave cavity sensor when sample is measured for 30 min (R^2^ = 0.967) and 6 min (R^2^ = 0.8955).

An aim of this process was to establish the minimum time in which a result with acceptable agreement to the EZ-Driploss measurement could be obtained. While a measurement time of 30 min is clearly favorable when compared to the current 24 h required of the EZ-Driploss test, it is of industrial value to reduce this time as much as possible. To this end, the captured data was analyzed with a view to understanding the point at which the correlation between the microwave cavity and EZ-Driploss measurements fell significantly. Referring to Figure 9, the maximum R^2^ value is obtained over the full 30 min measurement (R^2^ = 0.967). It is also noted that at 6 min, the R^2^ value (0.896) is still acceptable; below this measurement time the correlation drops significantly to a minimum of 0.401. A comparison of the data produced for 30 and 6 min measurements is evidenced in Figure 10.

While there is still some way to go to produce a commercially viable rapid non-invasive drip loss measurement system, this work demonstrates that it is possible to provide a measurement with good correlation to the existing industry standard EZ-Driploss test within 6 min. The correlation between the results evidenced in this paper exceeds that of other reported non-invasive systems such as X-ray or NIRS. Furthermore, microwave sensor systems are much cheaper by comparison, since the components to produce them are often based upon wireless electronics available in consumer devices (e.g., Wi-Fi, mobile phones, *etc.*). Concerns regarding safety are also alleviated since the system used in this work utilizes low power (<10 dBm) non-ionizing radiation.

## 6. Conclusions/Outlook

This paper presents a novel microwave cavity sensor for the measurement of drip loss, correlating results obtained from 24 pork loin samples against the widely used EZ-Driploss test, which typically takes 24 h to yield a result. It was shown that the sensor can provide a maximum correlation of R^2^ = 0.967 with a 30 min measurement time, or a weaker correlation of R^2^ = 0.896 with a 6 min measurement time. Not only does the sensor provide results in good agreement with the EZ-Driploss method, it demonstrates comparable, if not better, performance than reported alternative automated techniques, such as NIRS, X-ray and electrode based methods.

Future work in developing this technique could consider a number of directions, including the application of the technique to a broader spectrum of meat types (e.g., beef and lamb) in addition to considering the translation of the method for online use. Owing to the highly flexible nature of microwave spectroscopy, particularly in terms of the format of the sensor, the team foresees that it may be possible to identify and sort carcasses with high drip loss at an early stage post-slaughter. This would have significant implications for the industry in relation to meat production costs.
